# Prevalence of Hypertension in Adult Population of A Village of Nepal

**DOI:** 10.31729/jnma.4536

**Published:** 2019-08-31

**Authors:** Smriti Khanal, Krishna Rana, Milan Chandra Khanal, Astha Prasai, Anjila Pradhan, Manisha Shahi

**Affiliations:** 1 Deurali Primary Health Centre, Nuwakot, Nepal; 2 Shepherd College, New Baneshwor, Kathmandu, Nepal; 3 Himal Hospital, Gyaneshwor, Kathmandu, Nepal

**Keywords:** *blood pressure*, *hypertension*, *Nepal*

## Abstract

**Introduction:**

Hypertension is a cardiovascular disorder rapidly emerging as a major health problem in developing countries. Uncontrolled or poorly managed hypertension leads to several complications such as coronary heart disease, peripheral vascular disease and kidney disease. These complications account for approx 9.4 million deaths worldwide every year. Consequently, it is an urgent need for authorities to act upon this issue. This study was done to determine the prevalence of Hypertension in Deurali village of Nuwakot.

**Methods:**

A descriptive cross-sectional study was done in the Deurali Village of Nuwakot district from May 2019 to July 2019. Ethical approval was taken from the Ethical Review Board of Nepal Health Research Council. A simple random sampling method was applied. Blood pressure was measured on three occasions along with the use of a self-administered questionnaire. Descriptive statistical analysis was done.

**Results:**

The study showed the prevalence of hypertension among the adult population to be 20 (8.5%) [8.5%±1.83% at 95% CI]. Prevalence was found to be higher among female than male. Age group more than 60 were major sufferers of the condition. The study population consisted of the majority of Tamang community. Almost 213 (91%) participants were reported to have taken alcohol at some point in their life whereas only 104 (45%) were smokers.

**Conclusions:**

The prevalence of hypertension was found to be lower than the previous study done in similar settings.

## INTRODUCTION

Hypertension has dramatically risen as a global burden in recent years. Hypertension is a common health problem in developing countries and prevalence is currently rising steadily. When it comes to Nepal, the prevalence of hypertension in various parts varies between studies which ranged between 3.3% and 44.9%. The first hypertension survey in Nepal was done in 1982 by Mrigendra Samjhana Medical Trust (MSMT) and the prevalence rate was 6% according to the old WHO criteria 160/95.^1^ A repeat cross-sectional study done in a rural Kathmandu revealed that prevalence of Hypertension tripled from 6% in 1980 to 18% in 2006.^2^ A number of recent studies demonstrate that there is a direct relationship between blood pressure and cardiovascular events.^2^ The treatment of the high BP can reduce cardiovascular morbidity and mortality. Besides, non-communicable diseases including cardiovascular diseases are exerting enormous burden on life of poor and marginalized people reducing labour productivity and increasing out of pocket expenditure and ultimately creating more pressure on poor healthcare system and debilitating natural economy.^3,4^

This study aims to determine the prevalence of Hypertension in Deurali village of Nuwakot.

## METHODS

A descriptive cross-sectional study was conducted in Deurali Village of Nuwakot district of Nepal. After taking the ethical clearance from the Ethical Review Board, data was collected from 233 people of the village from May to July 2019. The sample size of this study was calculated using the formula,

 Sample size (n)= Z^2^ x pq/e^2^

     =(1.96)^2^ × 0.18 × (1-0.18) / (0.05)^2^

       =226

In Deurali Village, population (N): 3600

Adjusted Sample size: n/ 1+n-1/N

      = 212

Where,

Confidence Interval (CI)= 95% Margin of error (e)= 5% p= prevalence which was taken as 18% from the previous study done in Nepal^5^

q= (1-p)

Therefore, the calculated sample size was 212. Adding the 10% non-response rate, the sample size that was taken is 233.

Simple random sampling technique was applied. The list of the people living in ward no 3 (Deurali) was taken from ward office. A consecutive number was given to the list. Random number table was utilized to select the sample. People aged 18 years above and providing consent were included in the study. People aged below 18 years, not a resident of Deurali, pregnant lady, serious and debilitated patient were not included in the study. Consent paper was read out to the people who can not read and write for getting a thumbprint and informed consent paper were given for signature to those who can read and write. Then a self-administered questionnaire was read to them and according to their response, the form was filled. Aneroid Sphygmomanometer and Littmann Master Cardio Stethoscope were used to measure blood pressure in all participants.

Despite simple random sampling, the following bias could occur: Interviewer Bias, Recall Bias and Social Desirability Bias.

Data collected via self-administered questionnaire was kept in Microsoft Excel and then edited and checked. After that, the data was put in SPSS. Frequency, percentages were calculated for binary data after the normality of the data has been checked. The descriptive statistical analysis was done.

## RESULTS

**Figure 1.**
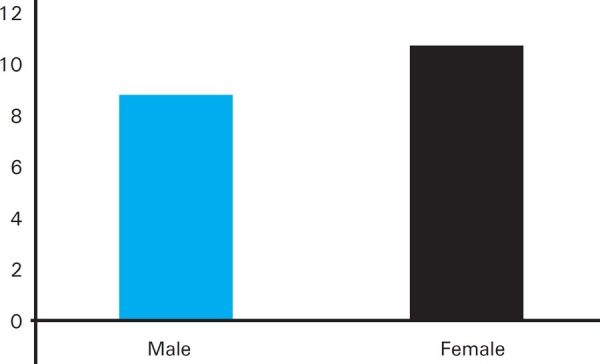
Genderwise prevalence of hypertension.

The period prevalence of hypertension among the adult population in Deurali village is 20 (8.54%) [8.54% ±1.83% at 95% CI]. Among 234 participants, there were 102 male and 132 female; hypertension was more prevalent among female than male (Figure 1).

**Figure 2.**
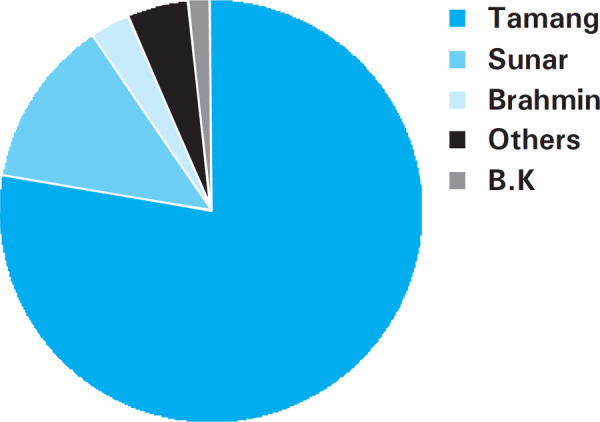
Caste Distribution.

Regarding socio-demographic characteristics, most of the participants were housewife 90 (38.5%) followed by farmer 58 (25%). Similarly, majority of the participants were Tamang followed by Sunar (Fig. 2).

Most of the participants were from age above 60 69 (30%) which was followed by 18-30 age group 63 (27%). In terms of literacy, most of the participants were illiterate 102 (43.5%) whereas only 21 (9%) studied till secondary level and only 10 (4.27%) had studied up to higher level. Most of the hypertensive participants were from age 60+ group 10 (50%) followed by age 46-59 age group 8 (40%).

Moving toward lifestyle patterns, 213 (91%) participants admitted that they have consumed alcohol at least once. In contrast, more than half of participants 130 (55%) stated that they have never done cigarette smoking in their entire life. Only one hypertensive patient told that he never consumed alcohol. Among all hypertensive patients, only one participant was not taking anti-hypertensive medications. Eleven hypertensive patients have left drinking habit after the diagnosis of their condition. Among 20 hypertensive participants, 9 (45%) participants’ blood pressure was not controlled with medications.

After blood pressure measurement, the highest BP recorded was 170/110 mm Hg and the lowest BP recorded was 90/60 mm Hg.

## DISCUSSION

In our study, the prevalence of hypertension is found to be 8.5% which, as compared to the previous study, is lower. Despite the drinking habits of the majority of the participants only 20 persons were having hypertension.

In a study done by Manandhar K et al, the prevalence of hypertension was 44.9% (47.75% in male and 42.73% in females).^6^ They found higher proportion of hypertensive cases in age > 65 years (55.49%) than in the age group <65 years (36.32%) which was similar to our study. A study done by Pandey et al. in 1981 showed that overall prevalence of hypertension to be only 5.98 % in a rural population in Nepal.^1^ However, a study conducted by Sharma et al in 2005 in the suburban area of Kathmandu, Nepal showed a very high prevalence of 42% among people aged above 50 years.^7^ The hilly geography of the location with people having to walk miles every day for household chores could be the reason behind lower prevalence in our study.

In our study, the prevalence of hypertension was found to be higher among female than male. Various studies done have shown that that hypertension is more prevalent in men compared to women whereas, some other studies showed female preponderance.^8–12^ The reason for more prevalence among female in our study could be attributed to lack of physical activities and dietary factors.

One of the risk factors for hypertension is increased age. In this study, the proportion of hypertensive participants increases as age advances. Several studies also reported that the prevalence of hypertension increased with age.^13,14^ Similarly, alcohol and smoking which are considered to be major modifiable risk factors for the development of hypertension were found to be practised in significant number in our study with almost 90% of the participants with drinking habits. The study done by Shrestha S et al also reflected the correlation of hypertension with smoking and alcohol habit.^15^

The limitation of our study is that the sample size taken cannot be generalized over the large area. The blood pressure measurement could have been biased due to inappropriate positioning and timing due to various household activities that need to be done by village person in time. Social desirability bias could have misled the information given.

## CONCLUSIONS

The prevalence of hypertension was found to be lower than the previous study done in similar settings. For better research findings in the future, the study involving large area with increased number of sample size is recommended.

## Conflict of Interest:

**None.**

## References

[1] Panday MR, Upadhyaya LR, Dhungel S, Pillaik K, Regmi HN, Neupane RP (1981). Prevalence of hypertension in a rural community in Nepal. Indian Heart J.

[2] Lee DS, Massaro JM, Wang TJ, Kannel WB, Benjamin EJ, Kenchaiah S (2007). Antecedent blood pressure, body mass index and the risk of incident heart failure in later life. Hypertension.

[3] Bhandari GP, Angdembe MR, Dhimal M, Neupane S, Bhusal C (2014). State of non-communicable diseases in Nepal. BMC Public Health.

[4] Abegunde DO, Mathers CD, Adam T, Ortegon M, Strong K (2007). The burden and costs of chronic diseases in low-income and middle-income countries. Lancet.

[5] Sharma SK, Ghimire A, Radhakrishnan J, Thapa L, Shrestha NR, Paudel N (2011). Prevalence of hypertension, obesity, diabetes and metabolic syndrome in Nepal. Int J Hypertension.

[6] Manandhar K, Koju R, Sinha NP, Humagain S (2012). Prevalence and Associated Risk Factors of Hypertension Among People Aged 50 years and more in Banepa Municipality Nepal. Kathmandu Univ Med J (KUMJ).

[7] Sharma SK, Dhakal S, Thapa L, Ghimire A, Tamrakar R, Chaudhary S (2013). Community based screening for chronic kidney disease, hypertension and diabetes in Dharan. JNMA J Nepal Med Assoc.

[8] Velazquez MO, Rosas PM, Lara EA (2002). Arterial hypertension in Mexico: results of the National Health Survey 2000. Arch Cardiol Mex.

[9] Joffres MR, Ghadirian P, Foder JG (1997). Awareness, treatment, and control of hypertension in Canada. Am J Hypertens.

[10] Stein AD, Stoyanovsky V, Mincheva V (2000). Prevalence, awareness, treatment and control of hypertension in a working Bulgarian population. Eur J Epidemiol.

[11] Sonmez HM, Basak O, Camci C (1999). The epidemiology of elevated blood pressure as an estimate for hypertension in Aydin, Turkey. J Hum Hypertens.

[12] Jenei Z, Pall D, Katona E The epidemiology of hypertension and its associated risk factors in the city of Debrecen, Hungary. Public Health.

[13] Tsai PS, Ke TL, Huang CJ, Tsai JC, Chen PL, Wang SY, Shyu YK (2005). Prevalence and determinants of prehypertension status in the Taiwanese general population. J Hypertens.

[14] Kunz I, Schorr U, Klaus S, Sharma AM (2000). Resting metabolic rate and substrate use in obesity hypertension. Hypertension.

[15] Shrestha S, Devkota R (2016). Prevalence of hypertension and its associated risk factors in a sub-urban area of central Nepal. Int J Community Med Public Health.

